# Implementation of a delirium assessment tool in the ICU can influence haloperidol use

**DOI:** 10.1186/cc7991

**Published:** 2009-08-10

**Authors:** Mark van den Boogaard, Peter Pickkers, Hans van der Hoeven, Gabriel Roodbol, Theo van Achterberg, Lisette Schoonhoven

**Affiliations:** 1 Department of Intensive care medicine, Radboud University Nijmegen Medical Centre P.O. box 9101, Internal post 685, Nijmegen, 6500HB, The Netherlands; 2 Department of Psychiatry, Radboud University Nijmegen Medical Centre, P.O. box 9101, Internal post 963, Nijmegen, 6500HB, The Netherlands; 3 Scientific Institute for Quality of Healthcare, Radboud University Nijmegen Medical Centre, Geert Grooteplein noord 21, Internal post 114, Nijmegen, 6525 EZ, The Netherlands

## Abstract

**Introduction:**

In critically ill patients, delirium is a serious and frequent disorder that is associated with a prolonged intensive care and hospital stay and an increased morbidity and mortality. Without the use of a delirium screening instrument, delirium is often missed by ICU nurses and physicians. The effects of implementation of a screening method on haloperidol use is not known. The purpose of this study was to evaluate the implementation of the confusion assessment method-ICU (CAM-ICU) and the effect of its use on frequency and duration of haloperidol use.

**Methods:**

We used a tailored implementation strategy focused on potential barriers. We measured CAM-ICU compliance, interrater reliability, and delirium knowledge, and compared the haloperidol use, as a proxy for delirium incidence, before and after the implementation of the CAM-ICU.

**Results:**

Compliance and delirium knowledge increased from 77% to 92% and from 6.2 to 7.4, respectively (both, *P *< 0.0001). The interrater reliability increased from 0.78 to 0.89. More patients were treated with haloperidol (9.9% to 14.8%, *P *< 0.001), however with a lower dose (18 to 6 mg, *P *= 0.01) and for a shorter time period (5 [IQR:2–9] to 3 [IQR:1–5] days, *P *= 0.02).

**Conclusions:**

With a tailored implementation strategy, a delirium assessment tool was successfully introduced in the ICU with the main goals achieved within four months. Early detection of delirium in critically ill patients increases the number of patients that receive treatment with haloperidol, however with a lower dose and for a shorter time period.

## Introduction

Delirium is a common psychiatric disorder in critically ill patients. It has an acute onset and combines cognitive and attention defects with a fluctuating consciousness [1]. It is associated with a prolonged intensive care and hospital stay and an increased morbidity and mortality [2-4].

Although there has been increasing interest in delirium in the past five years, standard screening of patients in daily practice is still not common, resulting in an underestimation of the problem. Previous studies showed that, without the use of a screening instrument, more than 60% of patients with delirium are missed by ICU nurses and more than 70% by physicians [5,6]. It can therefore be assumed that delirious patients are not sufficiently treated if they are not recognized. The incidence rate in critically ill patients varies between 11% and 87%, depending on the study design, methods for assessment, and differences in population [2,4,7-9].

Although there is no evidence that the use of a delirium assessment tool results in improvement of outcome, early recognition of delirium is important for adequate and early treatment. Therefore routine screening of patients is necessary. In addition, because of the fluctuating clinical signs and symptoms of delirium, screening should be performed at least once every 8 to 12 hours [10,11]. A delirium assessment tool should therefore be quick and easy to use with a high interrater reliability.

The Dutch guidelines *Delirium in the Intensive Care *recommends the screening of all ICU patients with a reliable and validated delirium screening instrument (van Eijk MJJ, Spronk PE, van den Boogaard MHWA, Kuiper MA, Smit EGM, Slooter AJC. Delirium op de Intensive Care, unpublished data), such as the intensive care delirium screening checklist (ICDSC) [12] or the confusion assessment method-ICU (CAM-ICU) [13].

The treatment of delirium is based on removing the underlying somatic disorder frequently combined with pharmacological therapy. Although there is no clear evidence that treatment improves the prognosis of delirious ICU patients [14], and haloperidol has significant side effects [15,16], haloperidol is the most commonly recommended pharmacological agent [17]. As screening will probably increase the number of patients diagnosed with delirium, it could also increase the use of haloperidol. In view of this, it is important to determine the effect of the implementation of a screening instrument on the use of haloperidol.

The first aim of our study was to evaluate our strategy for the implementation of the CAM-ICU. Therefore, the compliance with scoring of the CAM-ICU, the interrater reliability, and improvement in delirium knowledge of the nurses were used as indicators for successful implementation. We assumed that a larger number of delirious patients would be detected with the use of the CAM-ICU, in comparison with previous periods without the standard use of a screening tool. The second aim of our study was therefore to assess how the CAM-ICU influences the frequency and duration of haloperidol use, which may be considered to be a proxy for the delirium incidence and duration.

## Materials and methods

This study was conducted in the Radboud University Nijmegen Medical Centre, the Netherlands, a 960-bed university hospital that includes a level 3 (highest level) ICU with 40 beds divided over four adult wards and one paediatric ward. Annually 2000 to 2500 (cardiothoracic surgery, neurosurgical, medical, surgical, and trauma) patients are admitted.

The local Institutional Review Board of Arnhem-Nijmegen indicated that for this study no approval was required and no informed consent from patients was needed.

### Nurses and the implementation of the CAM-ICU

Although the ICDSC and the CAM-ICU are suitable delirium screening instruments, we preferred to implement the CAM-ICU above the ICDSC because of the higher sensitivity and specificity, and because the CAM-ICU is translated and validated in Dutch [18]. The CAM-ICU is an easy to perform assessment tool for ICU nurses, which consists of a two-step approach model [13] [see Additional data file 1]. Before the implementation of the CAM-ICU, identification of delirious patients was based on the judgement of the attending ICU physician, and a delirium screening instrument was not used. Due to the potential importance of unrecognised delirium, we decided that this should be changed to a situation where regular and systematic assessment of delirium was performed by ICU nurses with specific knowledge of delirium recognition. Therefore, we introduced the CAM-ICU as an instrument for early recognition of delirium and started with the implementation on all four adult ICU wards in December 2007.

Implementation of a delirium assessment tool in daily practice introduces an essential change for ICU nurses. As there is no single best method for implementing an innovation in all settings [19], it is important to identify potential barriers and facilitators in this particular setting. For a good adaptation of a delirium screening instrument it is important to tailor the implementation strategy to these facilitators and barriers [20]. Furthermore, support from the organisation and medical and nursing staff participation is important for a successful implementation [21].

Our implementation strategy [see Additional data file 2] was focused on potential barriers and facilitators for screening with the CAM-ICU (Table 1), which were identified during several, unstructured, interviews with the nursing and medical staff.

**Table 1:** Identified potential barriers and facilitators during interviews

Implementation barriers	Implementation facilitators
1. Lack of knowledge concerning delirium	1. Patient data management system
2. Inavailability of the assessment tool	2. Senior nurses
3. To fill in the delirium assessment tool on paper three times a day ('paperwork')	3. Support of medical and nursing staff
4. Time to perform the assessment	4. Delirium researcher

We integrated the CAM-ICU algorithm in our patient data management system, which is available at all bedside computers. Because of the fluctuating course of delirium every patient had to be assessed minimally once in every eight-hour shift, according to the CAM-ICU manual [22]. If the mental status changed after an assessment, an additional assessment had to be performed. Patients were excluded from screening when they had a Richmond agitation sedation score of -4 or -5 [13], were unable to understand Dutch, were severely mentally disabled, or suffered from a serious receptive aphasia. All necessary testing tools (attention screening pictures and disorganized thinking questions) were made available at every bed. The computer notified the nurse about the outcome of the CAM-ICU screening, that is, delirious or not.

Evidence-based interventions [23] included in the implementation strategy were: education; educational outreach visits; reminders and feedback; and leadership.

### Education and educational outreach visits

All ICU nurses were trained in the use of the CAM-ICU and performed a knowledge test prior to the training. The education consisted of a one-hour group training prior to the implementation of the CAM-ICU. During this training, information about delirium features, recognition, and delirium types was given. Furthermore, specific information was given about the CAM-ICU. We used educational material from the delirium website [22] such as the training video and the Harvard CAM-ICU flow sheet. We appointed 'delirium key-nurses', who received supplementary training, for further instruction and introduction of the CAM-ICU in their unit. In addition, posters with the Harvard CAM-ICU flow sheet were distributed to nurses and the medical staff. Also, the medical staff was informed about delirium and the CAM-ICU. Supplementary individual training on the job (by MvdB, and the 'delirium key-nurses') started one month after the implementation and was given whenever screening compliance and interrater reliability dropped below the stated aim. The focus during this training on the job was on the most common mismatches, that is feature 1A and 1B [see Additional data file 1]. Determination of the presence of cognitive function disturbances and the fluctuating nature of consciousness were the most difficult points for the ICU nurses. Individual problems with the assessment were addressed by focusing the training on the difficulties experienced during observations.

### Reminders and feedback

When a delirium assessment was not carried out, a pop-up appeared on the bedside computer as a reminder for the nurse. The CAM-ICU scoring rate, that is the screening compliance, and the interrater reliability were measured. The results were evaluated with the delirium key-nurses and the nursing staff, twice a week as parameters of a successful implementation. Feedback about results and performance of the CAM-ICU was supplied weekly by e-mail and during monthly clinical meetings.

### Leadership

The medical and nursing staff committed themselves to, and supported the implementation of the delirium assessment tool, as agreed upon during the information meeting and was reported during feedback of the key nurses. One project leader was responsible and supervised the implementation process (MvdB). Prior to the implementation, the CAM-ICU was introduced to the medical staff. Two months after the implementation, the presence of delirium became a standard part of the daily multidisciplinary meeting, in which all patients are discussed. All ICU wards were visited daily by the project leader to identify problems concerning the performance and compliance of the assessment tool and for personal or group feedback.

Chosen indicators of a successful implementation were: regular assessment of all ICU patients defined as a screening compliance of more than 80%; interrater reliability score of more than 0.80; and improvement of the level of knowledge concerning delirium.

The compliance was calculated as the percentage of performed assessments per day of the total number of assessments that should have been performed. Interrater reliability tests were performed several times during the first month after the implementation and twice a week during and after the training on the job period. For this the CAM-ICU score assessed by the ICU nurse was compared with the CAM-ICU score assessed by an expert psychiatric nurse (GR). The maximum period between the two assessments was one hour and patients were chosen randomly. Patients who were excluded from screening with the CAM-ICU were also excluded from the interrater reliability testing.

**Figure 1:**
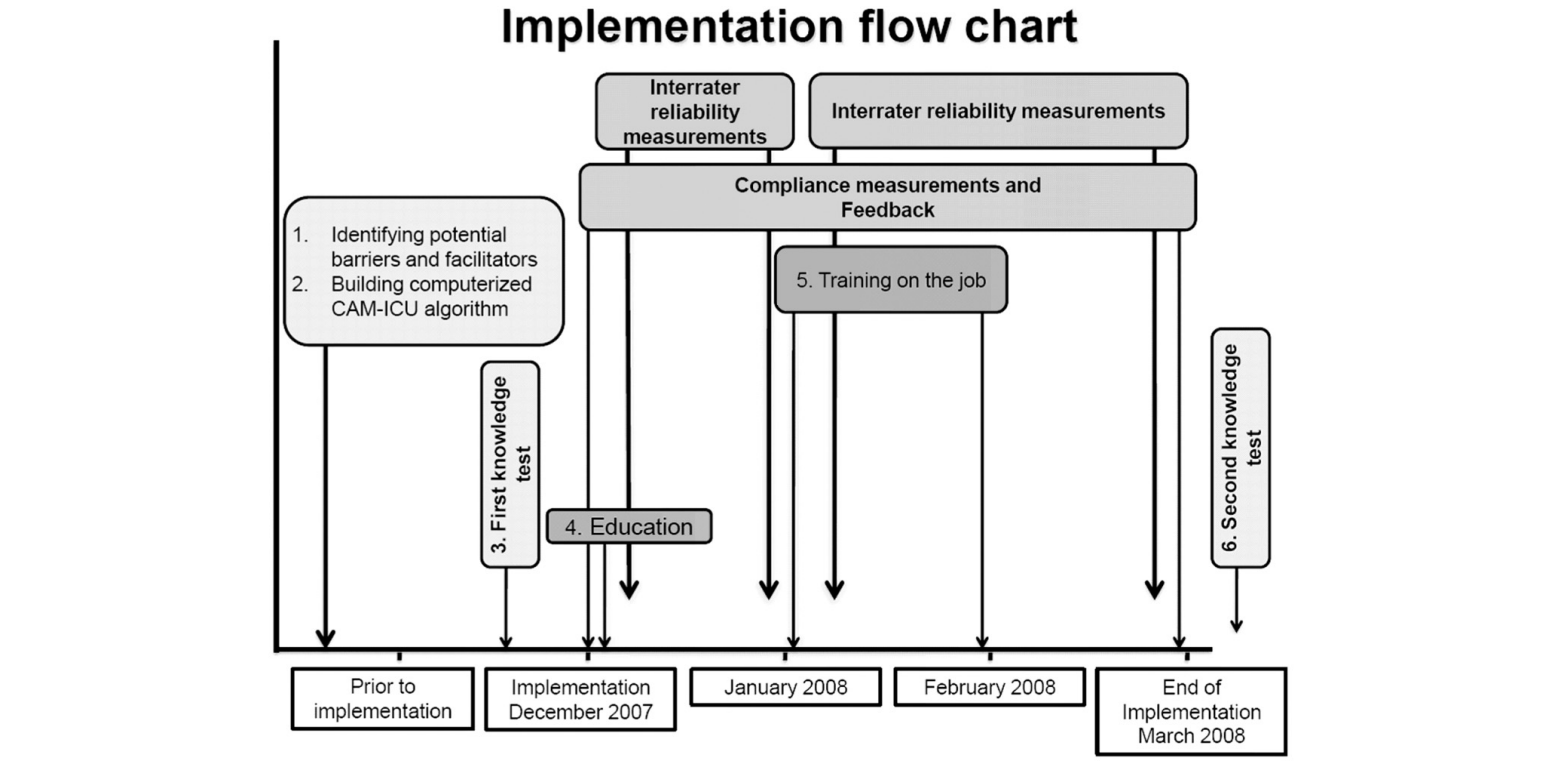
Implementation flow chart. CAM-ICU = confusion assessment method-intensive care unit.

We developed a non-validated written delirium knowledge test that had to be completed in 10 minutes prior to the delirium training and consisted of 10 mixed open and closed questions. A similar post-training test was performed four months later. 'Delirium knowledge' is expressed on a scale of 0 to 10. The implementation period started in December 2007 and ended in March 2008, after reaching the indicators of care improvement (Figure 1). The nursing staff consists of 140 nurses of which 18 (13%) were ICU nurses in training.

### The patients and haloperidol treatment

As delirium incidence rates before the use of the assessment tool were not available, we used the frequency of haloperidol use as a proxy for delirium incidence. Data of all patients who were treated with haloperidol are available through our patient data management system. As a general rule, in our ICU all patients diagnosed with delirium are treated with haloperidol and delirium is the only reason for prescribing haloperidol. The duration of haloperidol treatment was used as a proxy for the duration of the delirious period. For the incidence rate of a four-month period (March until June 2008) after the implementation, the CAM-ICU results were compared with the haloperidol use during the same period of the two previous years. We compared the total number of all consecutive patients treated with haloperidol, total days of treatment, and the total dose of administered haloperidol per patient and per day.

### Statistical analyses

All data analyses were performed with SPSS 16.0 (SPSS Inc., Chicago, IL, USA). Normally distributed data (demographic data, knowledge level, and the scorings rate) were tested parametrically (Student's t-test, repeated measurement analysis of variance). Data concerning the treatment with haloperidol were not normally distributed and were tested non-parametrically with the Friedman test and the Kruskal-Wallis one-way analysis of variance test. Interrater reliability of the outcome of screening, that is delirious or non-delirious, was calculated with the Cohen's Kappa statistic.

## Results

### Evaluation of implementation and nurses

In the first month of the implementation period the interrater reliability was 0.78 (n = 25, 95% confidence interval (CI): 0.5 to 1.0) and following intensive training on the job of almost all ICU nurses this increased to 0.89 (n = 47, 95%CI: 0.75 to 1.0).

In the first month after the implementation the compliance of screening with the CAM-ICU was 77% and increased significantly to 92% (repeated measurement analysis of variance, *P *< 0.0001) after four months. Scoring rate of the nurses at the pre-course delirium knowledge test was 6.2 ± 1.7 (n = 136) and increased significantly to 7.4 ± 1.2 (n = 122) four months later (Student's t-test, *P *= 0.0001).

### Haloperidol treatment and patients

With the exception of a small, but statistically significant difference in the Acute Physiology and Chronic Health Evaluation-II (APACHE-II) score, the demographic variables of the patients did not differ between the three years (Table 2). In the same period in 2006 and 2007, 13 (10%) and 20 (13%) patients per month were treated with haloperidol, respectively (Table 3). Following the implementation period, based on the CAM-ICU results, this increased significantly to 37 (23%) patients per month (*P *< 0.001) compared with the previous period without the use of the CAM-ICU. All patients who received haloperidol in the period after the implementation in 2008 were detected with the CAM-ICU as delirious patients. From these 147 delirious patients, 25 (17%) had a hyperactive type, 47 (32%) a hypoactive type, and 74 patients (50.3%) had a mixed-type delirium. During this period 641 patients were admitted of which 74 patients were excluded from CAM-ICU screening. The most frequent reason was sustained coma (49%). To compare the effect on the detected incidence before and after the implementation of the CAM-ICU, we used the total of 641 patients, because of the lack of information of the patients in the period before the implementation.

**Table 2:** Demographic variables of ICU-patients before and after implementation of CAM-ICU

Period	Prior to implementation March to June 2006	Prior to implementation March to June 2007	After implementation March to June 2008	*P *value
Number of patients	512	589	641	
Age	57.5 ± 16.4	58.9 ± 16.6	59.5 ± 15.6	N.S.
Gender (M/F)	339/173	370/219	409/232	N.S.
APACHE-II score	16.9 ± 7.0	17.1 ± 6.9	15.5 ± 6.5	0.0001
Length of stay on ICU in days (median (IQR))	1.3 (0.8 to 5.9)	1.0 (1 to 5)	1.0 (1 to 3)	N.S.
Admission type (n)				
Elective surgery	214 (42%)	283 (48%)	340 (53%)	N.S.
'Urgent surgery	106 (20%)	96 (16%)	76 (12%)	
Medical	192 (38%)	210 (36%)	225 (35%)	

All values are means ± standard deviation unless otherwise reported.

APACHE II = Acute Physiology and Chronic Health Evaluation II; CAM-ICU = confusion assessment method-intensive care unit; F = female; ICU = intensive care unit; IQR = interquartile range; M = male; N.S. = non-significant.

**Table 3:** Effect of the implementation of the CAM-ICU in 2008 on delirium treatment

	2006 (n = 512)	2007 (n = 589)	2008 (n = 641)	*P *value
Total numbers of delirious patients (%)	51 (10%)	79 (13%)	147 (23%)	< 0.0001
Number of delirious patients per month	13	20	37	< 0.0001
Total dose of haloperidol per patient (mg) n = total number of patients treated with haloperidol	18 (5 to 40) (n = 52)	12.5 (3 to 30) (n = 80)	6 (2 to 20) (n = 147)	0.01
Duration of treatment (days)	5 (2 to 9)	3 (2 to 9)	3 (1 to 5)	0.02

All values are medians (interquartile range) unless other reported. CAM-ICU = confusion assessment method-intensive care unit.

The median duration of treatment with haloperidol decreased from five (interquartile range (IQR) 2 to 9) to three days (IQR 1 to 5) after the implementation of the CAM-ICU (*P *= 0.02). The median total haloperidol dose per patient (during treatment) decreased from 18 mg (IQR 5 to 39.5) to 6 mg (IQR 2 to 19.5; *P *= 0.01).

## Discussion

In a relatively short period of four months, we successfully implemented a validated delirium assessment tool in our daily practice on the ICU. Following the implementation of the CAM-ICU, more patients were treated with haloperidol, but with a lower dose and for a shorter period of time when compared with the same period in the two previous years. Almost two times more delirious patients were detected with the use of the CAM-ICU. Our results indicate that successful implementation of the CAM-ICU is possible and, importantly, that this results in shorter and lower dosed haloperidol treatment.

### The implementation of the CAM-ICU

We feel that several aspects of our implementation strategy are responsible for this success. First, we used a multifaceted model with evidence-based interventions. Although we did not measure the effect of the separate interventions, previous studies showed that education and feedback with reminders are very effective interventions [23]. Second, it is important to focus the implementation strategy on potential barriers that can be expected in daily practice [19], which will differ from hospital to hospital and from ward to ward. We therefore gathered information about these potential barriers prior to the actual implementation. Based on this information, we used the facilitators of our organization and integrated the CAM-ICU in our patient data management system. Although it took some time to develop the integrated CAM-ICU, it was easier to use and included a reminder when the assessment had not been performed at the end of the shift. The key-nurses played an important role in supporting the group and therefore were pivotal. They were also particularly helpful in bedside training of the ICU nurses, their direct colleagues.

A final point of interest is the cooperation with the medical staff. We noticed that it is important that the CAM-ICU score is part of the daily evaluation of the patient and that it is also important to react adequately to a positive delirious score by treating the patient. Therefore, it is also important to inform the medical staff during the implementation (education) and give them regular feedback on the results of the implementation (compliance, interrater reliability, and delirium knowledge level). As these interventions are tailored to the barriers found in this study they should not be used as a blueprint for implementation but could serve as a guideline.

Although the CAM-ICU appears to be relatively simple to use and a relatively short training period should result in a reliable performance of the CAM-ICU [11,13], our study demonstrates that an intensive implementation strategy results in a further improvement of its performance. We aimed for a group interrater reliability score of at least 0.8, which can be considered a desirable [24] and attainable goal for the CAM-ICU [13]. Evidently, it is of utmost importance to test the reliability of the assessment by the ICU nurses, because a false-positive diagnosis may result in unnecessary treatment and vice versa. Therefore, in our view, it is necessary to perform interrater reliability tests and analyse the mismatches to be able to give adequate feedback. Unfortunately, and surprisingly, not much attention is given to this aspect in the literature and many new screening and treatment policies appear to be implemented without it.

Although a high interrater reliability is important for the performance of the CAM-ICU, a screening tool will only be effective when the compliance with its use is also high. Although we did not formally measure the nursing workload, it is clear that the screening of patients with the CAM-ICU results in some additional work for the nurses. Our experience is that the mean screening time of the patients with the CAM-ICU is two to five minutes, which is comparable with that mentioned by Ely and colleagues [13]. Based on a study by Soja and colleagues [25] we chose an 80% compliance with the CAM-ICU as a feasible and acceptable aim for a successful implementation. Scoring all patients three times a day during their whole stay on the ICU is hardly realistic. Moreover, an optimal compliance is unknown. We are convinced that the intensive feedback and support of the project leader and the medical and nursing staff played an important role in achieving a high compliance.

### Haloperidol treatment and patients

One could argue that haloperidol use is not a good proxy for the incidence of delirium because it is also used to treat other disorders such as serious psychoses, severe excitement, and anxiety [26]. However, these disorders are rarely observed in our ICU or not treated with haloperidol. In the case of agitation in patients without a protected airway we use a low dose of propofol, if necessary in combination with oxazepam. Therefore we are confident that in our ICU only delirious patients are treated with haloperidol and that the observed difference in haloperidol use between the compared treatment periods can only be attributed to differences in delirium detection rate.

Despite the fact that we found a higher incidence of delirious patients with the CAM-ICU than without the use of a screening instrument, the incidence in our population is low. A possible explanation is that the study was performed in all consecutive patients, with no selection of high-risk patient groups. Including patients that were admitted to our ICU following elective surgery may also partly explain why the APACHE II score is lower compared with other studies that reported higher APACHE II scores associated with a higher incidence of delirium [13,27,28].

It is assumed that the regular use of a delirium assessment tool results in a higher detection rate of delirious patients, especially patients with a hypoactive delirium. Naturally, this could result in more haloperidol use. Given the potential side effects of the drug, the absence of clear evidence that presence of hypoactive delirium is associated with poor patient outcome and that the use of a delirium assessment tool improves the outcome of the ICU patient, one might argue that an increase in haloperidol use is not desirable. On the other hand, an earlier and improved recognition of delirious patients may make it easier to treat the delirium with lower doses of haloperidol. To our knowledge, the influence of performing the CAM-ICU on the total amount of haloperidol used per patient has not been studied before. It appears plausible that, besides the earlier detection of delirious patients, also recovery from the delirious period could be detected earlier with the use of a delirium assessment tool. As a result, haloperidol treatment would be stopped earlier. Our data confirm these assumptions. It is also possible that the early treatment of delirium could result in shortening of the delirious period, but this assumption needs further study.

## Conclusions

Tailoring our implementation strategy to the needs of the ICU was successful. The main goals were achieved within a relatively short time. Early recognition of delirium with the CAM-ICU has become a standard component of daily care by the nurses in our ICU and contributes to the quality of care. In addition, early detection of delirium leads to lower dosage and shorter periods of haloperidol treatment in critically ill patients.

## Key messages

• Implementation of the CAM-ICU is feasible and results in a higher determination rate of delirium.

• When the CAM-ICU is used, more patients receive haloperidol, but in a lower dose and for a shorter period of time.

## Abbreviations

APACHE-II: acute physiology and chronic health evaluation-II; CAM-ICU: confusion assessment method-intensive care unit; CI: confidence interval; ICDSC: intensive care delirium screening checklist; IQR: inter quartile range.

## Competing interests

The authors declare that they have no competing interests.

## Authors' contributions

MvdB carried out the study, gathered all data, performed the statistical analysis, and drafted the manuscript. PP and LS supervised the conduct of the study and writing of the paper. HvdH and TvA corrected the manuscript. GR carried out the interrater reliability measurements. All authors read and approved the final manuscript.

## Supplementary Material

Additional data file 1 Appendix 1 confusion assessment method-intensive care unit (CAM-ICU) worksheet. Click here for file

Additional data file 2 Word file containing a table that lists the implementation strategy. Click here for file
