# Supplementary material for: Implementation of a delirium assessment tool in the ICU can influence haloperidol use
Source: Crit Care. 2009 Aug 10;13(4):R131. doi: 10.1186/cc7991 (PMC2750188; doi:10.1186/cc7991)
Supplement: Additional data file 1 — Appendix 1 confusion assessment method-intensive care unit (CAM-ICU) worksheet. [file cc7991-S1.doc]

**Appendix 1** CAM-ICU worksheet

| **Feature 1**: Acute Onset or Fluctuating Course  Positive if answer ‘ yes’ to either 1A or 1B | Positive | Negative |
| --- | --- | --- |
| **1A**: Is the pt. different than his/her baseline mental status?  Or  **1B**: Has the patient had any fluctuation in mental status in the past  24 hours as evidence by fluctuation on a sedation scale (e.g.  RASS) GCS, or previous delirium assessment | Yes | No |
| **Feature 2**: Inattention  Positive if either score for 2A or 2B is less than 8.  Attempt the ASE letters first. If pt. Is able to perform this test and the score is clear, record this score and move to Feature 3. If pt. Is unable to perform this test or the test score is unclear, then perform the ASE pictures. If you perform both tests, use the ASE pictures’ results to score the Feature | Positive | Negative |
| **2A: ASE letters:** record score (enter NT for not tested)  *Directions: Say to the patient, “I am going to read you a series of 10 letters. When you hear the letter ‘A’ indicate by squeezing my hand”. Read letters from the following letter list in a normal tone.*  SAVEAHAART  Scoring: errors are counted when patient fails to squeeze on the letter ‘A’ and when the patient squeezes on any letter other than ‘A’. | Score (out of 10):----------- | |
| **2B: ASE pictures:** record score (enter NT when not tested)  *Directions are included on the picture packets.* | Score (out of 10):------------ | |
| **Feature 3**: Disorganized thinking  Positive if the combined score is less than 4 | Positive | Negative |
| **3A:** Yes/No Questions  (Use either Set A or B, alternate on consecutive days if necessary):  **Set A** **Set B**  1. Will a stone float on water? 1. Will a leaf float on water?  2. Are there fish in the sea? 2. Are there elephants in the sea?  3. Does one pound weigh more 3. Do two pounds weight more  than two pounds? than one pound?  4. Can you use a hammer to 4. Can you use a hammer to cut  pound a nail? wood?  **Score_____**(patient earns 1 point for each correct answer out of 4)  **3B:** Command  Say to patient: “Hold up this many fingers”(examiner holds two fingers in front of patient) “Now do the same thing with the other hand”(not repeating the number of fingers). *If pt. Is unable to move both arms, for the second part of the command ask patient “Add one more finger”)  **Score­­­­­_____**(Patient earns 1 point if able to successfully complete the entire command) | Combined Score (3A=3B): ______ (out of 5) | |
| **Feature 4:** Altered level of consciousness  Positive if the actual RASS score is anything other than “0” (zero) | Positive | Negative |
| **Overall CAM-ICU** (Features 1 and 2 and either Feature 3 or 4): | Positive | Negative |

With permission of the authors.

Copyright © 2002, E. Wesley Ely, MD, MPH and Vanderbilt University, all rights reserved
