# Supplementary material for: Implementation of a delirium assessment tool in the ICU can influence haloperidol use
Source: Crit Care. 2009 Aug 10;13(4):R131. doi: 10.1186/cc7991 (PMC2750188; doi:10.1186/cc7991)
Supplement: Additional data file 2 — Word file containing a table that lists the implementation strategy. [file cc7991-S2.doc]

Textbox: Implementation strategy

**Interventions for the CAM-ICU implementation. We:**

1. made an inventory of potential facilitators and barriers of our organization
2. tailored the implementation strategy to the potential barriers and opportunities
3. set clear and feasible targets for a successful implementation
4. computerized the CAM-ICU algorithm in our system and made it user friendly
5. integrated reminders for screening in the computerized CAM-ICU
6. equipped every bed with all necessary tools for the assessment performance
7. appointed delirium ‘key-nurses’ for dissemination of delirium knowledge and assistance during the implementation
8. involved medical and nursing staff in the implementation
9. performed interrater reliability tests and provided extra training on the job on shortcomings encountered
10. provided regular feed-back on the progress of the implementation
